# Early mortality following ART initiation in HIV-infected adults and children in Uganda and Zimbabwe

**DOI:** 10.1186/1758-2652-13-S4-O37

**Published:** 2010-11-08

**Authors:** AS Walker, P Mugyenyi, P Munderi, J Hakim, AA Kekitiinwa, E Katabira, CF Gilks, C Kityo, P Nahirya-Ntege, K Nathoo, D Gibb

**Affiliations:** 1 MRC Clinical Trials Unit, London, UK; 2 Joint Clinical Research Centre, Kampala, Uganda; 3 MRC/UVRI Uganda Research Unit on AIDS, Entebbe, Uganda; 4 University of Zimbabwe Clinical Research Centre, Harare, Zimbabwe; 5 Pediatric Infectious Disease Centre, Mulago, Uganda; 6 Infectious Disease Institute, Makarere University, Kampala, Uganda; 7 Imperial College, London, UK; 8 University of Zimbabwe Medical School, Harare, Zimbabwe

## Purpose of study

Adults initiating ART in low-income countries have higher mortality in the first 3 months on ART than those in high-income countries, with more similar mortality risks after 6 months. However, the specific pattern of changing mortality risk after ART has not been investigated. It is also not known whether children initiating ART are at the same high risk of early mortality as adults in resource-limited settings.

## Methods

We used flexible parametric proportional hazards models to investigate how the risks of death vary over the first year on ART in adults (18+ years) from the DART trial and children (6 months-15 years) from the ARROW trial. We then estimated survival after ART initiation according to pre-ART CD4/CD4% and investigated the impact of age, sex and CD4/CD4% in multivariable models.

## Results

Similar changes in early mortality were observed in both adults and children. At all CD4/CD4%, mortality risk increased from enrolment to a maximum between days 30-45, then declined rapidly to day 180, then declining more slowly throughout the rest of the first year on ART. Estimated mortality 14, 30, 90, 180 and 365 days after ART initiation is shown in Table 1

**Table 1:** 

	DART	DART	DART	DART	ARROW	ARROW	ARROW	ARROW	ARROW	ARROW
Age (years)	18+	18+	18+	18+	4-15	4-15	4-15	0-3	0-3	0-3

pre-ART CD4/CD4%	0-49	50-99	100-149	150-199	0-49	50-99	100+	0-4%	5-9%	10%+

N	1106	784	759	661	131	56	552	27	87	348

Deaths in 1st year	103	36	23	17	14	2	7	2	4	9

Days after ART				Estimated	cumulative	mortality				

14	0.4%	0.1%	0.1%	0.1%	0.5%	0.1%	0.0%	0.4%	0.1%	0.1%

30	1.5%	0.6%	0.3%	0.3%	1.7%	0.5%	0.1%	1.5%	0.6%	0.3%

90	4.9%	2.1%	1.4%	1.1%	5.9%	2.3%	0.6%	5.2%	2.4%	1.3%

180	7.2%	3.4%	2.3%	1.8%	8.3%	3.5%	1.0%	7.4%	3.6%	2.0%

365	9.4%	4.5%	3.2%	2.5%	10.1%	4.5%	1.3%	9.1%	4.6%	2.6%

Pooling data across adults and children, after adjusting for CD4/CD4% group there was no evidence of an impact of age (p=0.29) or sex (p=0.17) on mortality during the first year on ART. There was also no evidence of a difference in mortality risk between those 4+ years with CD4<50 cells/mm3 and 0-3 with CD4%<5% (p=0.68), those 4+ years with CD4 50-99 and 0-3 with CD4% 5-<10% (p=0.48) or those 4+ years with CD4 100+ and 0-3 with CD4% 10%+ (p=0.24).

## Conclusions

Children do not have significantly poorer survival on ART than adults. However, children aged 4 years and over and adults with low CD4 have remarkably similar, and high, risks of mortality in the first 3 months after ART initiation compared to those with higher CD4. Children under 4 years with low CD4% are also at similar higher mortality risks.

